# Life as I know it

**DOI:** 10.4103/0019-5545.31610

**Published:** 2006

**Authors:** Mona Singh

I did not want to begin this story till I knew how it would end. Then on an ordinary day it came—the feeling of life was back. In that fleeting moment I knew that I would not have to end my story with a question mark. There is no background, no circumstance that shaped it. Nor were there any reasons, because there can be as many different backgrounds and reasons as there are individuals who have tales of depression to tell.

I will talk of a time when my insides craved for death. Not suicide—suicide would have been easier. But death makes you count each moment without any promise. When you look into a mirror you see a dead person living your life. Beyond that image, you are under heavy stillness—alive; craving for the life that was.

Within myself, I felt replaced by something I did not recognize. Probably in the manner of bewilderment experienced by a newborn who cannot understand his life, his breath, dreams, language, and the world.

I had not slept for six months. I did not stop thinking during that period. I wove a story which I lived and believed to be true—it was not. It was probably because of grief and disappointment, and the tendency to spend time alone, not wanting to share my weak moments with anyone else, that I disconnected myself from the world and escaped into a new space of imagination. When I finally emerged from my reverie I was not myself. There was a voidful absence. The story that I had been writing with my fingers typing endlessly for days was a story that none other could read.

That crashed my belief in everything. I could not trust my understanding of what was right or wrong, what was real and what was not, what existed and what did not, what was true and what was a fallacy. There was a complete sense of bafflement, disillusionment and betrayal.

With an aura of dejection, there was a sadistic peace in the nothingness. The sheer joy of being as well as the sheer agony of existence. And, on that ordinary day it came like the wind and slowly welcomed me back to something I thought I had lost forever. I still feel the hopelessness, the futility. But that window of a moment was enough to give me my answers. I know now that I will not just be a spectator to my life.

Human mind, once stretched by a new idea never regains its original dimension. If you wish to search the unexplained truths of life and a reality that is not visible, make a foray into your mind and it will not disappoint you. While you look for answers to questions beyond your reality in this intangible world or read the signs and patterns in the complexity, your dreams, your stories, your questions, your life—they all pass you by. While you look for the meaningless, you miss out on the meaningful. To mitigate your existence you roll into a state of obsessive thinking—that is the beginning of schizophrenia. Once the process begins, every little occurrence acts as a catalyst—as if the whole universe conspires against you. The belief in your truth becomes so overpowering that you reject all other truths to uphold this one, because this truth gives superiority and authority. When you can do anything, and be anything.

In this euphoric state your being explodes in an extreme state of interpretation, of language, of signs, of words—from people, television sets, from what is written, from what is not written, from the universe. The body becomes acutely aware of the energy patterns that pass through it—energies of a different being, as if possessed. You allow yourself to be someone else—a multiple personality. When you can hear the music in the air and dance to it like a madman, it feels like *nirvana.*

After that you are god or the messenger (read schizo-phrenic) receiving signs from Him.You have come to save the world. Some go public and proclaim themselves to the world. They either get laughed at, are ridiculed, or sent to an asylum. Others like me set to work in silence, and try to move mountains with the sheer power of the mind. We concoct our plots and strategies; and redesign the world with the speed of thought, and all sorts of ancient and modern sciences, and what not.

The obsession, the insomnia, the incessant activity of the brain, the hormonal imbalance causes a breakdown of the senses. The body reacts. You cannot taste food. You shut your eyes but cannot sleep. You begin to hallucinate. Things begin to change form. You hear the whole world singing along with you. Everyone seems to be talking about you. Someone keeps whispering your name.Then you see a sight so beautiful and sublime it's got to be heaven.

In the dark, your mother turns into a horse and you try to strangle her. The fright in her eyes strikes a certain emotion in you, a faint memory of something of the past. You want to stop thinking; but you cannot. You do not know how to. Your brain will not listen to you. It is relentless, cruel. You just want some sleep. Your sister whispers softly in your ear—baby, it's all your imagination. And for the first time, you hear her words, and believe in someone, other than yourself.

The six months seem like a moment—one which erased your past. Just like that. And you can feel no more what you were. Nor what you are. It is a sense of timelessness and statelessness.

Someone who loves us enough not to abandon us, has been feeding us, bathing us, cleaning us. Trying to keep us from killing ourselves; or killing them. Trying to put us to sleep—to understand our gibberish; our inane actions; and trying in every way to restore our sanity. While we were single-mindedly pursuing our goal to save the world, understand the zero, or find 15 Park Avenue, whatever it may be. I chose not to let my life be dictated by the experience of a few months. If you choose otherwise and allow yourself to relapse into the state you have emerged from—that is schizophrenia.

You need to be informed of your illness and become aware of your situation. Not everyone is privileged enough to have someone to help them at such a time in their lives; family and friends may not be educated about your condition and may not be willing to find out. Schizophrenia is difficult to understand and is usually diagnosed when enough damage has already been done. Many don't even have access to medical aid due to lack of awareness in spite of excellent practitioners in the field in private as well as government institutions.

I was lucky to have a family that cared for my sanity. I needed to first accept that something was going wrong, which is the most difficult thing to do. It may take a lifetime for some. But once you accept you open the door you had shut and can chose to be on either side of it. Of course it is not easy to accept that you are wrong, but you need to trust someone. You need to let someone help you when you can no longer help yourself. For once you need to let someone else make decisions for you. You need family and friends to give you strength when you have none. I wanted rest and I wanted cure. For that you need medicine and you need therapy. Many people who suffer from schizophrenia or depression do not want to take medicine or therapy and vehemently oppose it or do not find anyone to force it on them—that is the biggest hinderance to its cure. The medicines played a very vital role as they began to stop my mind from tripping and cut off my obsessive stream of thought which I was finding difficult to restrain on my own.

After this initial phase came the dead end of extreme depression, which I could not have handled without a psychiatrist. Someone who understood me and what I had been through and was now going through; who was like a friend when I found myself none to relate to. My psychiatrist had complete faith in me. She gave me time and she gave value to my words, my thoughts. She helped me understand myself and find a way out instead of imposing her guidance. She helped me to communicate with myself and with others. Most importantly, she helped to restore my balance and bring normalcy in my relations with others, with my family and friends who became overprotective, with myself as I had become cynical, angry, non-communicative and under-confident. Schizophrenia is an illness from which you can recover if you want to. Depression is something from which you can pull yourself out of if you want to.

There is no telling of the collateral damage that was done to my mind, body and soul. My memory blanks out and my brain tires of thoughts. My mind and body seem to stop functioning. For a long time I had to fight extreme intolerance and fear of people, crowds and conversations. Dreams were so vivid that I could not differentiate them from reality. Nightmares gave me insomnia. At times, I still feel like a child wanting to wail endlessly for no reason, to contort my face in careless childlike expressions, and to double up and sleep forever. There is temptation of returning to that silent unreasonable world and never resurfacing. It takes time to heal. But healing I am. That feeling of being at the edge all the time and being pulled back into that vortex is steadily dissipating. I have figured out formulae to deal with the bouts of extreme depression that make me feel suicidal. And most importantly, I take my medication regularly and discuss with my psychiatrist when I cannot deal with myself. Besides, I know this is nothing; people suffer much, much worse—disability, war, terrorism, disease, calamity, crime, abuse, trauma, discrimination, deprivation—and battle it to survive. It is the spirit that counts.

Mona Singh lives in Rohtak

